# Substrate utilization and energy expenditure pattern in sepsis by indirect calorimetry

**DOI:** 10.1186/s13054-020-03245-2

**Published:** 2020-08-31

**Authors:** Andrew Li, Amartya Mukhopadhyay

**Affiliations:** 1 Yong Loo Lin School of Medicine, National University of Singapore, Singapore; 2 Division of Respiratory and Critical Care Medicine, Department of Medicine, National University Hospital, Singapore; 3 Medical Affairs, Alexandra Hospital, Singapore

**Keywords:** Indirect calorimetry, Respiratory quotient, Sepsis, Metabolism, Energy expenditure

Temporal metabolic profiles in sepsis are altered by the hormonal changes related to reduced food intake before admission and fasting after mechanical ventilation (MV), followed by refeeding [1]. Animal models suggest that preterminal rats were *hypo*metabolic, compared to surviving animals [2]. This was further corroborated among septic patients, where higher metabolic rates and lower respiratory quotients (RQ) were observed [3]. However, longitudinal data using indirect calorimetry (IC) remain sparse. We aimed to determine the temporal trends of energy expenditure (EE) and RQ among septic patients receiving MV.

All MV septic adults in the intensive care unit of a tertiary hospital were screened between September 2018 and December 2019 with ethics approval (DSRB 2017/01001). Patients were excluded if they received MV < 3 days, dialysis, fraction of inspired oxygen > 0.6, and chest drain.

Patients demographics, anthropometric indices, outcomes, energy (target 25 kcal/kg/day) and protein (target 1.5 g/kg/day) delivery, propofol dose, and glucose infusions were collected. We initiated enteral feeding within 24 h of MV commencement. IC data collected (RQ, oxygen consumption, carbon dioxide production, and total EE) using Carescape B650 (GE Healthcare, USA), standardized for temperature, barometric pressure, and humidity, were obtained post-intubation (baseline), within 2 h of feeding initiation and thereafter, daily up to 5 days or till extubation, whichever was earlier. A steady state of 30 min was mandated to ensure IC data validity.

Categorical and continuous variables were reported as proportion and mean (SD) or median (IQR), respectively. Comparisons of medians were performed using Mann–Whitney *U* test. All statistical tests were two-tailed. *P* < 0.05 was considered significant. To determine metabolism status, we followed the methods by Giovannini and Fried [2, 3]. *Hyper*- and *hypo*metabolism were determined by positive and negative EE when compared to the resting state by Harris-Benedict equation. To understand substrate utilization, we followed the RQ trends. A balanced diet’s RQ is approximately 0.8. We divided the cohort into RQ ≤ 0.8 (lipid as predominant substrate) and > 0.8 (carbohydrate as predominant substrate). STATA 14 (Stata Corp, College Station, TX, USA) was used.

Table 1 displays the baseline characteristics between survivors (*n* = 20) and non-survivors (*n* = 14). Median age and BMI were 63.5 (24–73) years and 22.5 (20.4–22.7) kg/m^2^, respectively. Mean APACHE II 27.7 (7.8), SOFA 14.3 (2.7), and mNUTRIC 6.2 (1.5) scores were noted.

**Table 1:** Comparison between survivors and non-survivors

	Survivors (*n* = 20)						Non-survivors (*n* = 14)					
Demographics												
Age, median (IQR) (years)	63.5 (24–72.5)						63.5 (24–74)					
Male (%)	12 (60%)						12 (85.7%)					
BMI, median (IQR) Kg/m^2^	22.23 (15.4–22.8)						22.74 (18.61–22.07)					
Clinical severity												
APACHE II, mean (SD)	27.5 ± 6.1						27.9 ± 9.3					
SOFA, mean (SD)	13.8 ± 2.8						15.1 ± 2.5					
mNUTRIC, mean (SD)	6.25 ± 1.61						6.21 ± 1.25					
SGA, median (IQR)	6 (3–6)						5 (1–5)					
Vasopressor use (%)	12 (60%)						11 (78.6%)					
Time to initiate feeding, median (range), hours	8.16 (3–45.1)						6.62 (3–23.25)					
Comorbidities												
Diabetes mellitus (%)	10 (50%)						4 (28.6%)					
Ischemic heart disease (%)	5 (25%)						4 (28.6%)					
Cerebrovascular accident (%)	5 (25%)						1 (7.1%)					
Chronic liver disease (%)	3 (15%)						1 (7.1%)					
Chronic kidney disease (%)	7 (35%)						3 (21.4%)					
Malignancy (%)	2 (10%)						3 (21.4%)					
Connective tissue disease (%)	5 (25%)						4 (28.6%)					
Diagnosis												
Pneumonia	8 (40%)						8 (57.1%)					
Line sepsis	2 (10%)						1 (7.1%)					
Other sites of sepsis	10 (50%)						5 (35.7%)					
Details of calories and macronutrient delivered												
	Total	Day 1	Day 2	Day 3	Day 4	Day 5	Total	Day 1	Day 2	Day 3	Day 4	Day 5
Calories delivered, median, IQR (kcal)	4655.45 (3439.96–5716.79)	821.01 (384.25–999.65)	845.40 (669.03–1102.08)	1072.22 (815.25–1329.05)	953.37 (817.38–1281.80)	1139.75 (1028.50–1421.00)	4600.76 (3754.91–6067.63)	590.71 (330.82–896.00)	1038.65 (637.48–1584.00)	1143.54 (808.75–1620.66)	941.25 (753.12–1419.75)	1063.03 (76.25–1490.29)
Carbohydrates delivered, median, IQR (g)	498.50 (379.50–646.00)	51.58 (34.07–98.77)	106.87 (69.52–117.94)	117.93 (96.86–145.8)	117 (109.33–129.6)	126.22 (98.28–159.69)	482.00 (400.00–581.00)	35.21 (24.95–83.02)	101.83 (62.24–129.6)	103.53 (86.65–131.53)	107.56 (86.4–125.95)	107.56 (93.79–132.53)
Lipid delivered, median, IQR (g)	190.50 (158.00–294.00)	23.43 (10.59–52.17)	55.61 (19.02–69.36)	43.2 (34.38–69.33)	43.2 (31.20–67.00)	57.29* (47.52–69.33)	154.50 (109.00–282.00)	19.38 (7.60–28.62)	36.47 (26.13–51.84)	35.91 (23.10–53.72)	34.62 (24.66–71.02)	29.95* (25.01–55.95)
Protein delivered, median, IQR (g)	323.74 (261.78–372.05)	44.92 (25.49–66.47)	61.17 (47.01–79.98)	74.88 (55.2–86.84)	66.36 (30.33–77.80)	70.25 (62.59–91.15)	279.33 (110.16–398.17)	34.32 (34.32–17.41)	69 (30.78–76.48)	66.8 (47.66–86.14)	61.91 (42.80–90.78)	77.68 (43.22–88.32)

*Abbreviations*: *IQR* interquartile range, *BMI* body mass index, *APACHE* Acute Physiology And Chronic Health Evaluation, *SOFA* sequential organ failure assessment, *mNUTRIC* modified Nutrition Risk in Critically ill, *SGA* Subjective Global Assessment, *IC* indirect calorimetry

*p = 0.02

The metabolic profiles differed between survivors and non-survivors (Fig. 1a). Both groups had negative energy balance during fasting state. Survivors transitioned to a *hyper*metabolic state following feeding initiation, achieving positive energy balance. Non-survivors remained *hypometabolic* despite feeding.

**Fig. 1:**
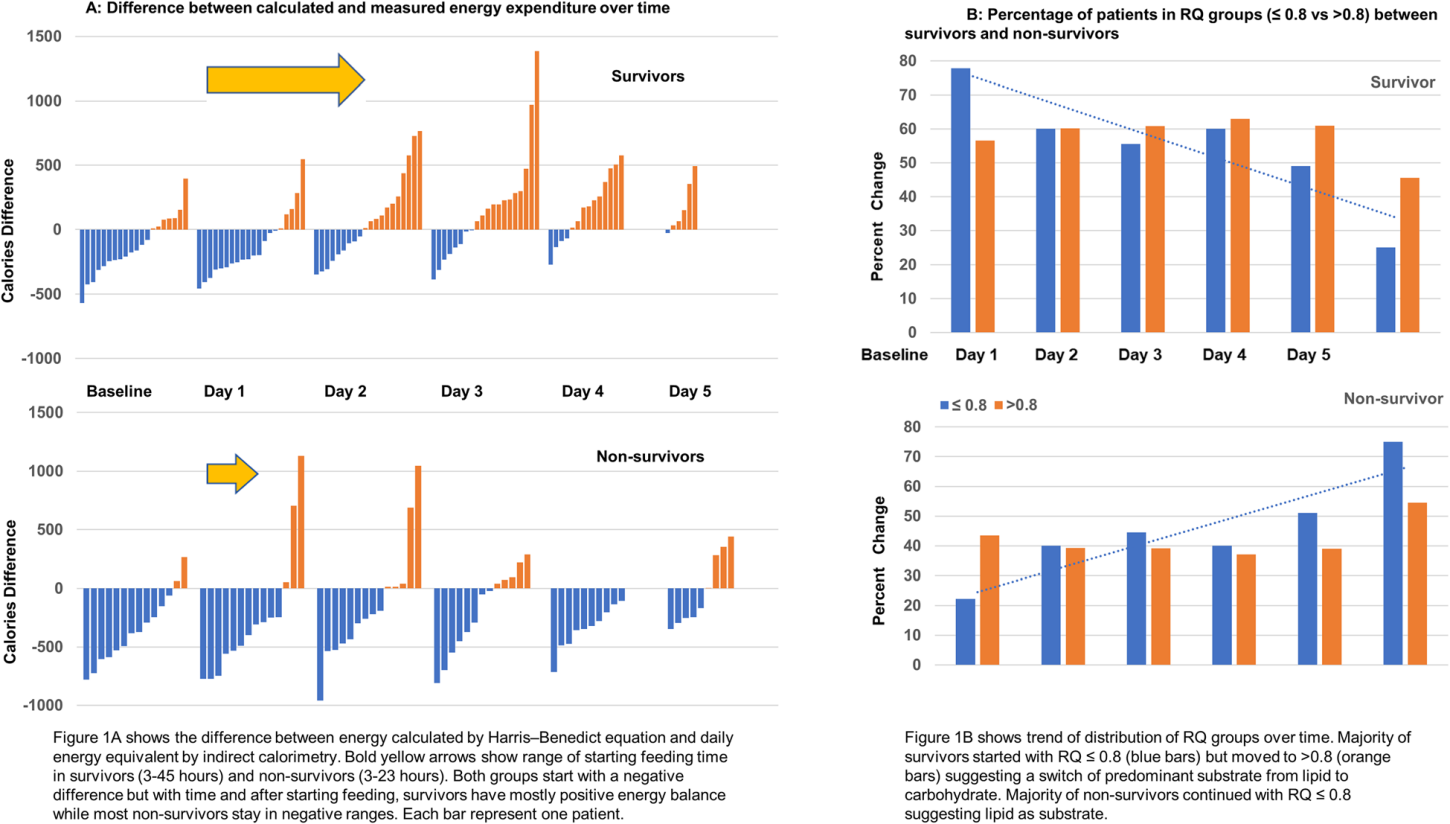
**a** Difference between calculated and measured energy expenditure over time. **b** Percentage of patients in RQ groups (≤ 0.8 vs > 0.8) between survivors and non-survivors

Most patients’ initial RQ were ≤ 0.8. Survivors transitioned into > 0.8 following feeding initiation, suggesting a change to carbohydrate metabolism. Most non-survivors RQ remained ≤ 0.8 (Fig. 1b). There was no difference in the daily energy and macronutrients delivered between survivors and non-survivors (Table 1).

Our study advances the understanding of energy balance and substrate utilization in sepsis. During fasting, low insulin with elevated counter-regulatory hormones promotes lipolysis; muscle glycogen is depleted at an exponential rate greater than athletes running marathons [4]. The predominant energy substrate switches from carbohydrates to lipids—the hallmark of fasting physiology. This explains the low RQ in early sepsis, when patients are preferentially utilizing lipids (RQ ≤ 0.8) during permissive underfeeding [5]. The *hype*rmetabolic state and inability for non-survivors to transit to carbohydrate utilization suggest ongoing debilitating mitochondrial dysfunction, consistent with associated multi-organ failure [6]. However, whether adjusting the feeding types and regimen to alter these patterns and improve outcomes remain unknown.

In conclusion, EE and substrate utilization patterns in early sepsis differ between survivors and non-survivors. Survivors assume higher EE and used carbohydrate as the main substrate while non-survivors have lower EE and predominantly utilized lipid.

## Data Availability

The datasets used and/or analyzed during the current study are available from the corresponding author on reasonable request.

## Abbreviations

MV: Mechanical ventilation
RQ: Respiratory quotient
EE: Energy expenditure
IC: Indirect calorimetry
