# Correction to: Substrate utilization and energy expenditure pattern in sepsis by indirect calorimetry

**DOI:** 10.1186/s13054-020-03391-7

**Published:** 2020-11-24

**Authors:** Andrew Li, Amartya Mukhopadhyay

**Affiliations:** 1 Yong Loo Lin School of Medicine, National University of Singapore, Singapore, Singapore; 2 Division of Respiratory and Critical Care Medicine, Department of Medicine, National University Hospital, Singapore, Singapore; 3 Medical Affairs, Alexandra Hospital, Singapore, Singapore

## Correction to: Critical Care (2020) 24:535 10.1186/s13054-020-03245-2

Following publication of the original article [1], the authors reported a misalignment error of the x-axis in Fig. 1b; in addition, there were two typos and two formatting errors. The revised Fig. 1b and revised text is indicated hereafter. The changes have been highlighted in **bold typeface**.

**Fig. 1:**
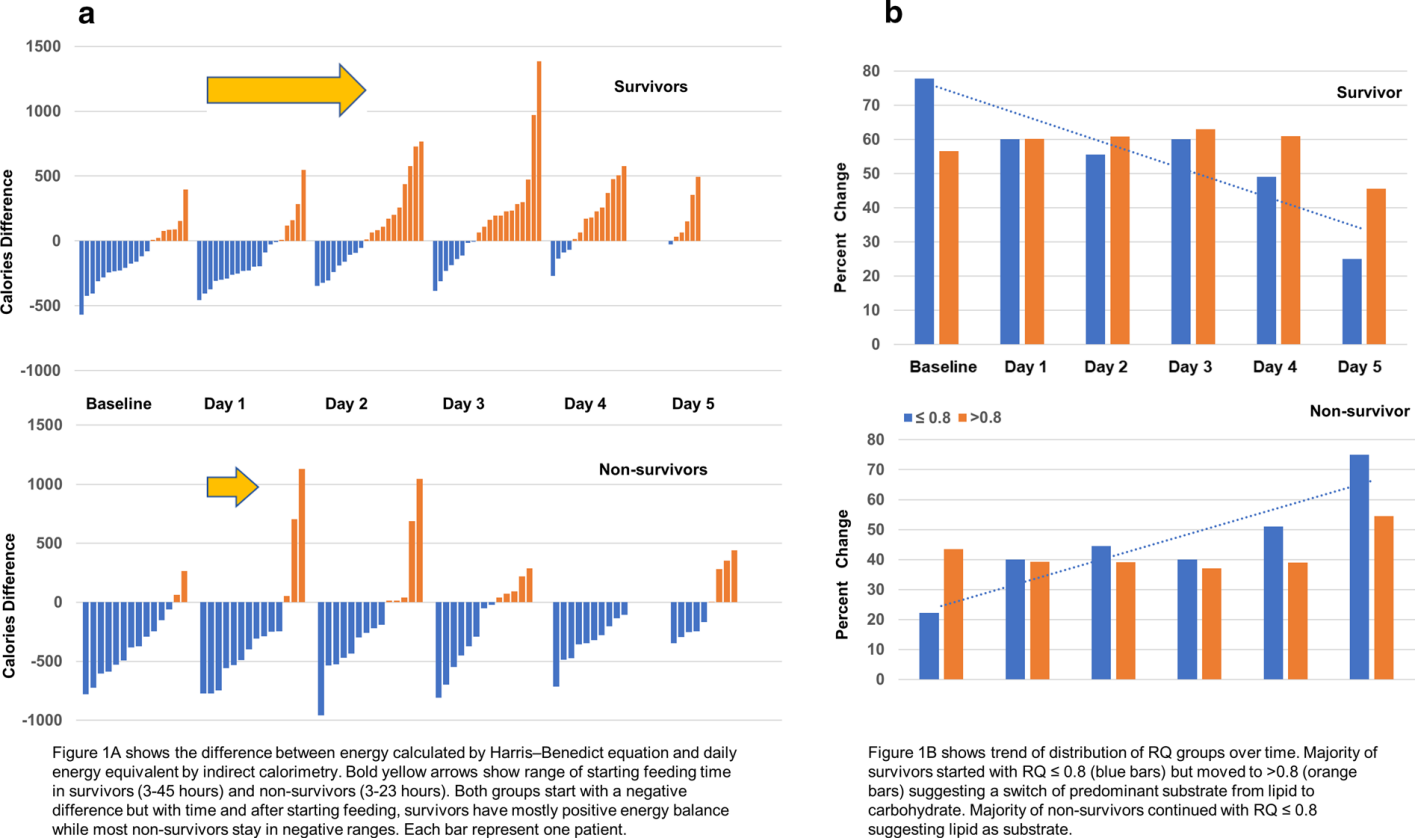
**a** Difference between calculated and measured energy expenditure over time. **b** Percentage of patients in RQ groups (≤ 0.8 vs. > 0.8) between survivors and non-survivors

The sentence currently reads:

The metabolic profiles differed between survivors and non-survivors (Fig. 1a). Both groups had negative energy balance during fasting state. Survivors transitioned to a *hyper*metabolic state following feeding initiation, achieving positive energy balance. Non-survivors remained *hypometabolic* despite feeding.

The sentence should read:

The metabolic profiles differed between survivors and non-survivors (Fig. 1a). Both groups had negative energy balance during fasting state. Survivors transitioned to a *hyper*metabolic state following feeding initiation, achieving positive energy balance. Non-survivors remained *hypo***metabolic** despite feeding.

The sentence currently reads:

Our study advances the understanding of energy balance and substrate utilization in sepsis. During fasting, low insulin with elevated counter-regulatory hormones promotes lipolysis; muscle glycogen is depleted at an exponential rate greater than athletes running marathons [4]. The predominant energy substrate switches from carbohydrates to lipids—the hallmark of fasting physiology. This explains the low RQ in early sepsis, when patients are preferentially utilizing lipids (RQ ≤ 0.8) during permissive underfeeding [5]. The hypermetabolic state and inability for non-survivors to transit to carbohydrate utilization suggest ongoing debilitating mitochondrial dysfunction, consistent with associated multi-organ failure [6]. However, whether adjusting the feeding types and regimen to alter these patterns and improve outcomes remain unknown.

The sentence should read:

Our study advances the understanding of energy balance and substrate utilization in sepsis. During fasting, low insulin with elevated counter-regulatory hormones promotes lipolysis; muscle glycogen is depleted at an exponential rate greater than athletes running marathons [4]. The predominant energy substrate switches from carbohydrates to lipids—the hallmark of fasting physiology. This explains the low RQ in early sepsis, when patients are preferentially utilizing lipids (RQ ≤ 0.8) during **relative** underfeeding [5]. The ***hypo***metabolic state and inability for non-survivors to transit to carbohydrate utilization suggest on-going debilitating mitochondrial dysfunction, consistent with associated multi-organ failure [6]. However, whether adjusting the feeding types and regimen to alter these patterns to improve outcomes remain unknown.

This has now been included in this correction article.
